# Assessing Seasonal Effects on Identification of Cultivation Methods of Short–Growth Cycle *Brassica chinensis* L. Using IRMS and NIRS

**DOI:** 10.3390/foods13081165

**Published:** 2024-04-11

**Authors:** Xing Liu, Kai Fan, Yangyang Lu, Hong Zhao, Qinxiong Rao, Hao Geng, Yijiao Chen, Karyne Maree Rogers, Weiguo Song

**Affiliations:** 1 Institute for Agro-Food Standards and Testing Technology, Shanghai Academy of Agricultural Sciences, Shanghai 201403, China; liuxinglyg@126.com (X.L.); fankai@saas.sh.cn (K.F.); yyanglz@163.com (Y.L.); hbmyzh@126.com (H.Z.); qinxiongrao@163.com (Q.R.); genghao0128@163.com (H.G.); cx1213097626@163.com (Y.C.); 2 Shanghai Service Platform of Agro-Products Quality and Safety Evaluation Technology, Shanghai 201403, China; 3 National Isotope Centre, GNS Science, 30 Gracefield Road, Lower Hutt 5040, New Zealand; 4 Institute of Agro-Product Safety and Nutrition, Zhejiang Academy of Agricultural Sciences, Hangzhou 310021, China

**Keywords:** *Brassica chinensis* L., seasonal effect, stable isotope, NIRS, cultivation method, PLS-DA

## Abstract

Seasonal (temporal) variations can influence the *δ*^13^C, *δ*^2^H, *δ*^18^O, and *δ*^15^N values and nutrient composition of organic (ORG), green (GRE), and conventional (CON) vegetables with a short growth cycle. Stable isotope ratio mass spectrometry (IRMS) and near-infrared spectroscopy (NIRS) combined with the partial least squares-discriminant analysis (PLS-DA) method were used to investigate seasonal effects on the identification of ORG, GRE, and CON *Brassica chinensis* L. samples (*BC*s). The results showed that *δ*^15^N values had significant differences among the three cultivation methods and that *δ*^13^C, *δ*^2^H, and *δ*^18^O values were significantly higher in winter and spring and lower in summer. The NIR spectra were relatively clustered across seasons. Neither IRMS-PLS-DA nor NIRS-PLS-DA could effectively identify all *BC* cultivation methods due to seasonal effects, while IRMS-NIRS-PLS-DA combined with Norris smoothing and derivative pretreatment had better predictive abilities, with an 89.80% accuracy for ORG and BCs, 88.89% for ORG and GRE *BC*s, and 75.00% for GRE and CON *BC*s. The IRMS-NIRS-PLS-DA provided an effective and robust method to identify BC cultivation methods, integrating multi-seasonal differences.

## 1. Introduction

Organic agriculture is based on environmentally friendly, product-safe, sustainable, and comprehensive agricultural practices, and is becoming a popular choice for global agricultural development. Furthermore, increasing awareness of health and environmental concerns among consumers is prompting them to pay more attention to organic products. Global organic market sales reached 134.8 billion euros in 2022 [1]. Countries actively promote organic farming methods while adopting relatively neutral and market-oriented policies based on their own circumstances. For example, China was ranked the third-highest producer of organic products in 2022, with a global market share of 9.2%. China’s government has also proposed to improve green agricultural standard and systems by strengthening the certification management of green food, organic products, and agricultural products with geographical indication status [2], sending strong signals that green agriculture is an important form of high-quality and sustainable development for Chinese agriculture. Green agriculture, defined by China National Standard GB/T 33761-2017 [3] and Agriculture Industry Standard NY/T 391-2021 [4], is a blend of organic and conventional agriculture, which primarily reduces the need for chemical fertilizers and pesticides.

As the societal demand for sustainable agriculture grows, the authenticity of organic and green foods has become an urgent issue due to profit-driven practices such as false advertising and misleading labels [5]. Several effective analytical methods have been used to ensure their authenticity, primarily including stable isotope analysis [6], spectral analysis [7], and chemical analysis [8]. In these methods, stable isotopes (*δ*^13^C, *δ*^15^N, *δ*^2^H, and *δ*^18^O) can objectively reflect climatic conditions (temperature, humidity, light intensity, and precipitation), soil composition, and agricultural input (fertilizers, pesticides, etc.) information for plant agricultural products [9]. For instance, *δ*^13^C values are indicative of photosynthesis pathways (C3, C4, or CAM) utilized by plants but are also influenced by light intensity, humidity, and environmental pollutants (car emissions) [9]. *δ*^15^N values offer information about plant nutrient sources and agricultural fertilization practices. Organic fertilizers (such as animal-derived manures and plant composts formed of non-leguminous products) typically exhibit relatively high *δ*^15^N values, distinguishing organic cultivation methods from green or conventional farming practices [9,10,11]. *δ*^2^H and *δ*^18^O values are used to identify different irrigation water sources and are influenced by rainfall, local temperatures, latitude, altitude, and distance from the sea [9,12]. Moreover, near infrared spectroscopy (NIRS) in spectral analysis mainly captures essential vibrational and rotational stretching details related to the hydrogen bonds (C-H, N-H, O-H, and S-H) of nutritional compositions of agricultural products and offers chemical-free, rapid, and non-destructive advantages in analyzing the composition of agricultural products [13].

Both stable isotope analysis and NIRS have been employed as powerful tools for the identification of organic, green, and conventional agricultural products based on their growing fertilizer types, climate environment, and nutritional component variations arising from different cultivation methods [11,14]. However, plant isotopes and NIR spectra can be altered by interannual climatic variations and seasonal effects, potentially affecting the accuracy of identifying cultivation methods [15,16]. This is particularly pronounced for crops with short growth or maturation periods (<60 days). Short–growth cycle leafy vegetables are more sensitive to changes in precipitation or temperature, causing variability in their chemical compositions and absorption characteristics of various elements [17]. However, seasonal effects on the stable isotopes and NIR spectra of short–growth cycle leafy vegetables need to be further investigated, along with the identification of short–growth cycle leafy vegetables cultivation methods using stable isotopes and NIRS.

Therefore, this study aimed to investigate the influence of seasonal variations on the *δ*^13^C, *δ*^15^N, *δ*^2^H, and *δ*^18^O values and NIR spectra of short–growth cycle and year-round planted *Brassica chinensis* L. (*BC*) under various cultivation methods. Furthermore, IRMS or/and NIRS data combined with chemometrics were used to ensure the authenticity of *BC* cultivation methods despite seasonal variations.

## 2. Materials and Methods

### 2.1. Sample Collection and Preparation

In this study, 175 *BC* samples comprising 63 organic (ORG) samples (defined by China National Standard GB/T 19630-2019) [18], 44 green (GRE) samples, and 68 conventional (CON) samples were collected between September 2020 and September 2021 from Shanghai vegetable farms. Samples collected from September 2020 to November 2020 were classified as autumn (mean temperature 19.6 °C), from December 2020 to February 2021 as winter (mean temperature 7.4 °C), from March 2021 to May 2021 as spring (mean temperature 16.9 °C), from June 2021 to August 2021 as summer (mean temperature 27.5 °C), and from September 2021 as autumn-repeat (autumn-re) (mean temperature 26.7 °C) (Table 1). Autumn-re samples were used as interannual samples to verify seasonal effects and identification models.

*BC* samples were prepared as reported in our previous paper [16]. Briefly, about 2.5 kg of each vegetable was collected, rinsed with deionized water to remove soil or dust, pulped using a blender, frozen at −18 °C for 6 h, and freeze-dried at −54 °C for at least 72 h. The dried samples were ground into a fine and uniform powder with a particle size less than 0.15 mm. The powder was stored in a desiccator to prevent the absorption of local atmospheric water and preserve the weakly reflected exchangeable and non-exchangeable *δ*^2^H and *δ*^18^O signatures from the different cultivation methods and seasonal effects.

### 2.2. Stable Isotope Analysis

The *δ*^13^C, *δ*^15^N, *δ*^2^H, and *δ*^18^O values of samples were determined using a Flash IRMS elemental analyzer (EA) interfaced to a DELTA V Advantage isotope ratio mass spectrometry system (IRMS, Thermo Fisher Scientific Inc., Bremen, Germany) using similar methods outlined in Liu et al. [19]. In C/N mode, the oxidation and reduction furnace temperatures of the EA were set at 980 °C. High purity helium was used as the carrier gas with a flow rate of 180 mL/min. High purity CO_2_ and N_2_ were used as the reference gases with flow rates of 60 mL/min. About 1.6 mg samples were weighed into tin capsules for *δ*^13^C and *δ*^15^N analysis with an 80% dilution ratio of CO_2_ produced by these samples during analysis. In H/O mode, the EA pyrolysis temperature was set at 1380 °C. High purity helium was used as the carrier gas with a flow rate of 100 mL/min. High purity CO and H_2_ were used as the reference gases, also at a flow rate of 100 mL/min. About 0.3 mg samples were weighed into silver capsules for *δ*^2^H and *δ*^18^O analysis with a 40% dilution ratio of H_2_ and a 60% dilution ratio of CO produced by these samples during analysis. Isotope ratios were calculated using the following Equation (1):

(1) $$X(‰)=\left(\frac{R_{sample}}{R_{standard}}-1\right)$$

where X represents *δ*^13^C, *δ*^15^N, *δ*^2^H, or *δ*^18^O; *R*_sample_ denotes the abundance ratio of heavy isotope against light isotope, e.g., ^13^C/^12^C, ^15^N/^14^N, ^18^O/^16^O, or ^2^H/^1^H; *R*_standard_ is the reference standard isotope ratio. Reference materials included USGS40 (*δ*^13^C = −26.389 ± 0.042‰, *δ*^15^N = −4.5 ± 0.1‰), USGS90 (δ^13^C = −13.75 ± 0.06‰, δ^15^N = +8.84 ± 0.17‰), and USGS91 (*δ*^13^C = −28.28 ± 0.08‰, *δ*^15^N = +1.78 ± 0.12‰) for *δ*^13^C and *δ*^15^N values; IAEA-603 (δ^18^O = −2.37‰ ± 0.04‰), USGS90 (*δ*^18^O = 35.90 ± 0.29‰), and USGS91 (δ^18^O = 21.13 ± 0.44‰) for *δ*^18^O values; and USGS54 (δ^2^H = −150.4 ± 1.1‰), USGS90 (*δ*^2^H = −13.9 ± 2.4‰), and USGS91 (*δ*^2^H = −45.7 ± 7.4‰) for *δ*^2^H values. A sample of *BC* was chosen as a quality control measure and included as a working standard; it was added after every 10 unknown samples. Instrumental precision was lower than ±0.1‰ for *δ*^13^C, ±0.2‰ for *δ*^15^N, ±2.0‰ for *δ*^2^H, and ±0.5‰ for *δ*^18^O.

### 2.3. NIRS Analysis

NIR spectra were collected using a Nicolet iS50 Fourier transform near-infrared spectrometer (Thermo Fisher Scientific, Waltham, MA, USA) with an integrating sphere mode and an InGaAs detector. The spectral range was from 10,000 cm^−1^ to 4000 cm^−1^, spectral resolution was set at 8 cm^−1^, scan time was 32, and an internal blank was used as the reference for the measurements. The powdered samples were thoroughly mixed before each scan and then placed in a rotating sample cup and scanned three times. NIR spectral data were captured using OMNIC 9 software and stored in absorbance format. An averaged spectrum, generated from the three replicate analyses, contained 1557 variables and was used for both the calibration and validation sets. The laboratory temperature was maintained at a constant 25 °C throughout the analysis period.

### 2.4. Statistical Analysis and Chemometrics Methods

One-way analysis of variance (ANOVA) was applied to evaluate and compare differences in the *δ*^13^C, *δ*^15^N, *δ*^2^H, and *δ*^18^O values of *BC*s attributed to seasonal effects using Matlab R2020a software (MathWorks, Natick, MA, USA). Boxplots created in Microsoft Office Excel 365 (Microsoft, Redmond, WA, USA) were employed to visually represent the differences between the four isotopes across different *BC* cultivation methods or seasons.

The spectral data underwent Norris smoothing and derivative (NSD) treatment prior to modeling, which aimed to reduce or eliminate random baseline shifts, light scattering, and noise interferences, ensuring that only useful information was incorporated into the spectral signal [20]. NSD pretreatment consists of smoothing involving parameters ‘s’ and ‘g’, where ‘s’ represents the number of data in one segment and ‘g’ is the number of data in one gap, and a derivative containing the first or second derivative. Before building the models, the Kennard–Stone (KS) algorithm was used to divide the samples into calibration set (75%) and validation set (25%) [21]. That is, the samples were selected one by one based on the furthest distance from each other using Euclidean distance, thus dispersing them across the multivariate space. Partial least squares-discriminant analysis (PLS-DA) [22] was utilized to identify the *BC* cultivation methods using Matlab R2020a software and SIMCA 14.1 software (Umetrics, Umeå, Sweden). K-fold cross validation (CV) was applied to determine the optimal number of latent variables (optLVs). The optLVs were used to build a calibration model of PLS-DA by sensitivity (SE), specificity (SP), area under curve (AUC), and classification accuracy for evaluation (see Supplementary Materials S1 for the equations of SE, SP, and classification accuracy) [23], and then a validation model was employed to predict the remaining samples (25%). The predictive ability of the model was assessed by an accuracy.

## 3. Results and Discussion

### 3.1. Overall and Seasonal Isotopes of Different BC Cultivation Methods

**Figure 1:**
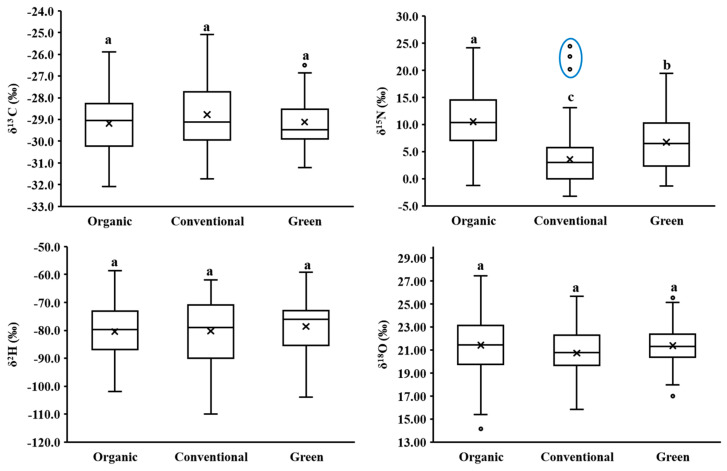
Stable isotope values of *BC* samples from different cultivation methods where “×” represents mean values; “-” represents median values; and “°” represents suspected outliers. Different lowercase letters among different cultivation methods indicate a significant difference at the *p* < 0.05 level.

There were differences in the *BC* stable isotopes determined for different cultivation methods (Figure 1). ORG *BC* had the highest overall mean *δ*^15^N (10.50 ± 6.20‰) and *δ*^18^O (21.42 ± 2.85‰) values, coupled with the lowest overall mean *δ*^13^C (−29.17 ± 1.36‰) and *δ*^2^H (−80.46 ± 9.68‰) values. Conversely, CON *BC* exhibited the highest overall mean *δ*^13^C (−28.78 ± 1.48‰) and the lowest overall mean *δ*^15^N (3.58 ± 5.33‰) and *δ*^18^O (20.73 ± 2.29‰) values, and GRE *BC* had the highest overall mean *δ*^2^H (−78.61 ± 9.60‰) values. Only the *δ*^15^N values showed a significant difference (*p* < 0.05) resulting from all the study samples collecting from Shanghai farms within a radius of around 40 km and experiencing similar climatic influences throughout the year. ORG, GRE, and CON *BC*s have different fertilization requirements. Specifically, ORG *BC* exclusively uses organic fertilizers (GB/T 19630-2019) [18], GRE *BC* is permitted to use chemical fertilizers in appropriate amounts (GB/T 33761-2017 and NY/T 391-2021) [3,4], and CON *BC* does not have significant restrictions on the use of chemical fertilizers.

Commonly, the organic fertilizers used in the cultivation of ORG and GRE *BC*s are animal manures or plant composts which undergo denitrification, promoting the volatilization of the light stable isotope ^14^N fraction, enhancing residual ^15^N in the fertilizer [24] and resulting in higher ORG and GRE *BC*s *δ*^15^N values compared to CON *BC*. However, if the storage and fermentation time of organic fertilizer, especially for animal manures, is short and denitrification has not occurred, or if the organic fertilizer is plant-based (legume), the *δ*^15^N values of organic vegetables may not be so high [25]. This effect might explain the low *δ*^15^N values (around 0‰) of some ORG *BC* samples (Figure 1). The study also found three CON *BC* samples with *δ*^15^N values exceeding 20‰ (Figure 1, blue circle), possibly due to the fact that these samples were from farms transitioning from conventional to organic cultivation. Slight *δ*^13^C, *δ*^2^H, and *δ*^18^O variations among *BC* cultivation methods were most likely due to the different fertilizer types and seasonal effects (temperature, light intensity, and precipitation), resulting in differences in carbon cycling, photosynthetic efficiency, and water use efficiency [9,26,27].

The *δ*^13^C, *δ*^15^N, *δ*^2^H, and *δ*^18^O values among ORG, GRE, and CON *BC* varied across different seasons (Table 2). There were no significant differences in seasonal mean *δ*^13^C and *δ*^2^H values among the three cultivation methods of *BC*, possibly indicating that the photosynthetic efficiency and water use efficiency of *BC* under different cultivation methods were similar in each season, given their similar growing locality in Shanghai. Seasonal mean *δ*^15^N values of *BC* also varied similarly among the three cultivation methods, following the sequence ORG > GRE > CON. Significant differences in *δ*^15^N values were observed between ORG and CON *BC*s during winter, summer, and autumn-re (*p* < 0.05). CON *BC*s exhibited significant differences compared to ORG and GRE *BC*s (*p* < 0.05) in spring. No significant differences were observed in autumn, possibly due to fertilizer type variations and the two outlier values (20.20‰ and 22.53‰) of CON *BC*s in autumn. ORG *BC*s had the highest seasonal mean *δ*^18^O value (23.37 ± 2.61‰) in spring and were significantly different from CON *BC*s (21.65 ± 1.50‰) (*p* < 0.05). This possibly arose from the enhancement of soil permeability and water retention caused by organic fertilizer application and the improvement of metabolic activity due to higher daily temperatures in late spring, leading to more positive ^18^O enrichment in *BC* tissue from H_2_^18^O and C^18^O_2_ [9,27,28].

The results confirmed that the *δ*^15^N values, mainly influenced by fertilizer type, characterized the three cultivation methods of *BC*. There were significant differences in some *δ*^15^N and *δ*^18^O values among different *BC* cultivation methods in a single season, indicating that different fertilizers were used for *BC*s grown under the same cultivation method. Moreover, *BC*s were also influenced by seasonal factors, such as temperature, light intensity, and precipitation, and required further investigation.

### 3.2. Seasonal Isotopes for Each BC Cultivation Method

**Figure 2:**
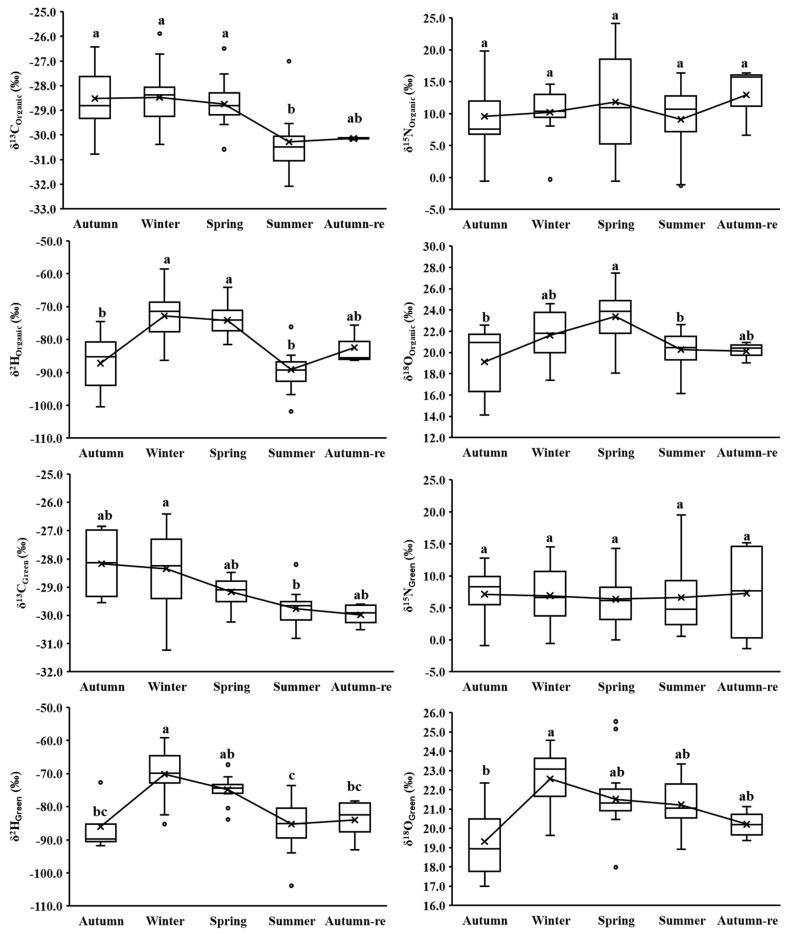

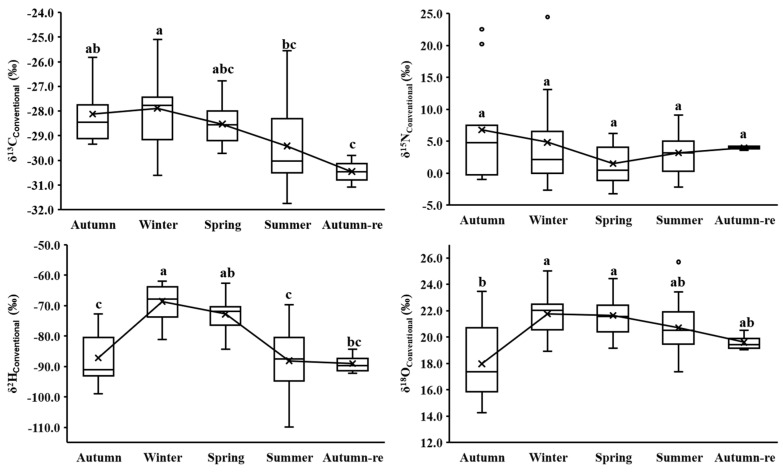
Stable isotopes of different *BC* cultivation methods based on seasonal time series where “×” represents mean values; “-” represents median values; “°” represents suspected outliers. Different lowercase letters among different seasons indicate a significant difference at the *p* < 0.05 level.

The stable isotopes of different *BC* cultivation methods showed distinct variations based on seasonal time series (Figure 2). Mean *δ*^13^C values for each season of organic (*δ*^13^C_Organic_), green (*δ*^13^C_Green_), and conventional (*δ*^13^C_Conventional_) *BC*s were higher in winter. A significant difference between winter and summer (*p* < 0.05) was noted, due to seasonal variations in temperature and light intensity influencing photosynthetic efficiency [9,27]. In summer, *BC*s exhibited vigorous photosynthesis, preferentially absorbing a higher proportion of lighter ^12^CO_2_ from the atmosphere, leading to lower *δ*^13^C values in *BC* tissue. Mean *δ*^15^N values for different *BC* cultivation methods showed no significant seasonal differences, as each cultivation method had specific fertilizer treatments. Each seasonal mean *δ*^2^H and *δ*^18^O value of different *BC* cultivation methods exhibited a similar trend across seasons (Figure 2). In winter and spring, the three cultivation methods of *BC*s had more positive mean *δ*^2^H and *δ*^18^O values compared to summer and autumn. There were significant *δ*^2^H and *δ*^18^O value differences between winter and/or spring and autumn, as well as between summer and autumn-re (*p* < 0.05). This trend was contrary to the usual expectations for precipitation, where *δ*^2^H and *δ*^18^O values are more positive in summer and more negative in winter [12,29]. However, it was consistent with the seasonal *δ*^2^H and *δ*^18^O variation in local atmospheric precipitation and the irrigation water source from the Yangtze River [30], which are significantly influenced by the distinct monsoon system and local topography around Shanghai [31,32,33]. In summer, precipitation in Shanghai, originating from the ocean, undergoes isotopic fractionation due to evaporation and condensation during a long transport process, leading to ^2^H and ^18^O depletion. In winter, the vapor from nearby water bodies, serving as a primary source of humidity, moisture content, and precipitation for Shanghai, typically shows relatively higher isotopic ratios [31,32].

These results suggest that seasonal changes can affect the *δ*^13^C and *δ*^15^N values of short–growth cycle *BC* under each cultivation method due to variable temperatures, light intensity, and fertilizer types. Additionally, seasonal effects can impact the *δ*^2^H and *δ*^18^O values through changes in precipitation and/or irrigation water sources. Therefore, when identifying cultivation methods for short–growth cycle vegetables, it is essential to investigate these seasonal effects to ensure that the developed model exhibits high applicability and accuracy across different seasons.

### 3.3. PLS-DA Isotope Models to Identify BC Cultivation Methods

Among the overall stable isotopes of different *BC* cultivation methods, only the *δ*^15^N values were significantly different among ORG, GRE, and CON *BC*s (Figure 1). The three CON *BC* outliers with high *δ*^15^N values (Figure 1, blue circle) came from farms transitioning from conventional to organic cultivation and could not represent the typical values of CON *BC*. After excluding these outliers, 80.95% of ORG *BC* had *δ*^15^N values above 6‰, while 43.18% of GRE *BC* and 81.54% of CON *BC* were below 6‰. The ORG and CON *BC*s could be well distinguished, but GRE *BC* overlapped both ORG and CON *BC*s due to the use of both fertilization methods. There were no significant differences in the overall mean *δ*^13^C, *δ*^2^H, and *δ*^18^O values of *BC* among different cultivation methods, as well as their seasonal mean *δ*^13^C and *δ*^2^H values. However, stable isotopes of individual *BC*s still exhibited variations within cultivation method classes due to seasonal variations in temperature, light intensity, and precipitation. A supervised PLS-DA method (IRMS-PLS-DA) was used to investigate these differences and attempt to improve the identification accuracy for ORG, GRE, and CON *BC*s (Table 3 and Table S1), especially for GRE *BC*. Moreover, the three outliers were included in the modeling data in order to ensure the universality of PLS-DA identification models.

**Figure 3:**
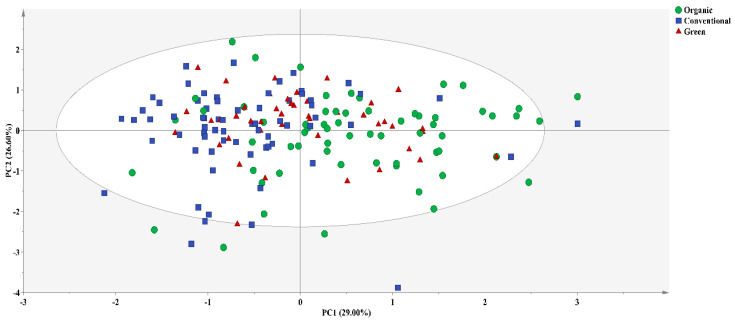
The first two principal component score plots of PLS-DA for different *BC* cultivation methods using IRMS.

The first two principal component score plots of PLS-DA (Figure 3) revealed that most ORG *BC*s could be effectively distinguished from CON *BC*s, but GRE *BC*s exhibited significant overlap with both ORG and CON *BC*s. Therefore, the discriminant model of the three cultivation methods was established in pairs. The calibration model accuracy for ORG and CON *BC*s was 77.55%, and the validation accuracy was 75.76%, with 24 ORG *BC*s being misclassified as CON *BC*s and six CON *BC*s as ORG *BC*s, possibly due to variations in fertilizer types and the comprehensive effects of seasonal variations and fertilizer types. For instance, some ORG *BC*s (*n* = 11) with *δ*^15^N values below 5‰ might have utilized plant-based (legume) organic fertilizers or the inadequate fermentation of manures [25], while some transitioning CON *BC*s only utilized organic manures. Moreover, some misclassified ORG *BC*s with *δ*^15^N values higher than 5‰ might have been influenced by seasonal variations in temperature, light intensity, and precipitation [9,26,27], and some misclassified CON *BC*s might be attributed to increased environmental protection by farmers, resulting in higher organic fertilizer use [34]. The highest misclassification rate occurred in summer (*n* = 10), probably due to higher levels of photosynthesis, water evaporation, and transpiration decreasing the differences between ORG and CON *BC*s during the summer [9,27] (Figure 2 and Table 3). The variable importance in projection (VIP) order used in the PLS-DA model was *δ*^15^N > *δ*^13^C > *δ*^18^O > *δ*^2^H, consistent with the stable isotope variations observed between ORG and CON *BC*s (Figure 1). 

The accuracy of the PLS-DA for the calibration model of ORG and GRE *BC*s was 71.25%, and for the validation model it was 51.85%, with 30 GRE *BC*s being misclassified as ORG *BC*s and six ORG *BC*s as GRE *BC*s, possibly attributed to smaller differences in *δ*^15^N values between ORG and GRE *BC*s (Figure 1), as ORG *BC*s used only organic fertilizers while GRE *BC*s could use both organic and chemical fertilizers (GB/T 33761-2017 and NY/T 391-2021). Summer *BC*s (*n* = 12) still demonstrated the highest misclassification rate, which may also be due to a combination of different fertilizer effects and seasonal variations [24,26]. The VIP order of the ORG and GRE *BC*s PLS-DA model was *δ*^15^N > *δ*^2^H > 1 > *δ*^13^C > *δ*^18^O, highlighting the significance of *δ*^15^N and *δ*^2^H values in distinguishing between GRE and ORG *BC*s. This order aligned with the overall stable isotope variations observed between ORG and GRE *BC*s (Figure 1). Specifically, ORG *BC*s showed the highest overall mean *δ*^15^N values (10.50 ± 6.20‰) and the lowest overall mean *δ*^2^H values (−80.46 ± 9.68‰), while GRE *BC*s demonstrated the highest overall mean *δ*^2^H values (−78.61 ± 9.60‰).

The calibration model of the GRE and CON *BC*s PLS-DA achieved an accuracy of 73.81%, with a validation accuracy of 53.57%. The number of misclassifications for GRE *BC*s (*n* = 30) was six times higher than that for CON *BC*s (*n* = 5), and misclassifications remained most pronounced during the summer, primarily due to the distinctive fertilization strategies employed by GRE *BC*s coupled with seasonal effects on the physiological and biochemical reactions of *BC*s [24,26]. The VIP order of the PLS-DA model was *δ*^15^N > *δ*^2^H > 1 > *δ*^18^O > *δ*^13^C, suggesting that fertilizer type and the irrigation water source influenced by seasonal precipitation were important modeling variables [8], and aligning well with the overall stable isotope variations between GRE and CON *BC*s (Figure 1). Specifically, GRE *BC*s exhibited significantly higher *δ*^15^N values and the highest *δ*^2^H values compared to CON *BC*s.

The results indicated that, despite incorporating the differences in four stable isotopes of individual *BC*s, the *BC* cultivation methods’ identification rates using PLS-DA models were not superior to those achieved by using only *δ*^15^N values, due to the influence of variations in fertilizer types and seasonal effects. Furthermore, the accuracy of identifying GRE *BC*s still needed to be improved. Overall, the study showed that *BC*s exhibited the highest misclassification rate in summer, resulting from the combination of high temperatures, intense sunlight, and frequent precipitation, leading to vigorous growth and the blurring of stable isotope differences among ORG, GRE, and CON *BC*s. We confirmed that seasonal factors had important impacts on the stable isotopes of *BC*s grown using different cultivation methods, consequently impacting the ability to clearly distinguish different *BC*s’ cultivation methods by isotopes alone.

### 3.4. NIR Spectra to Identify BC Cultivation Methods

**Figure 4:**
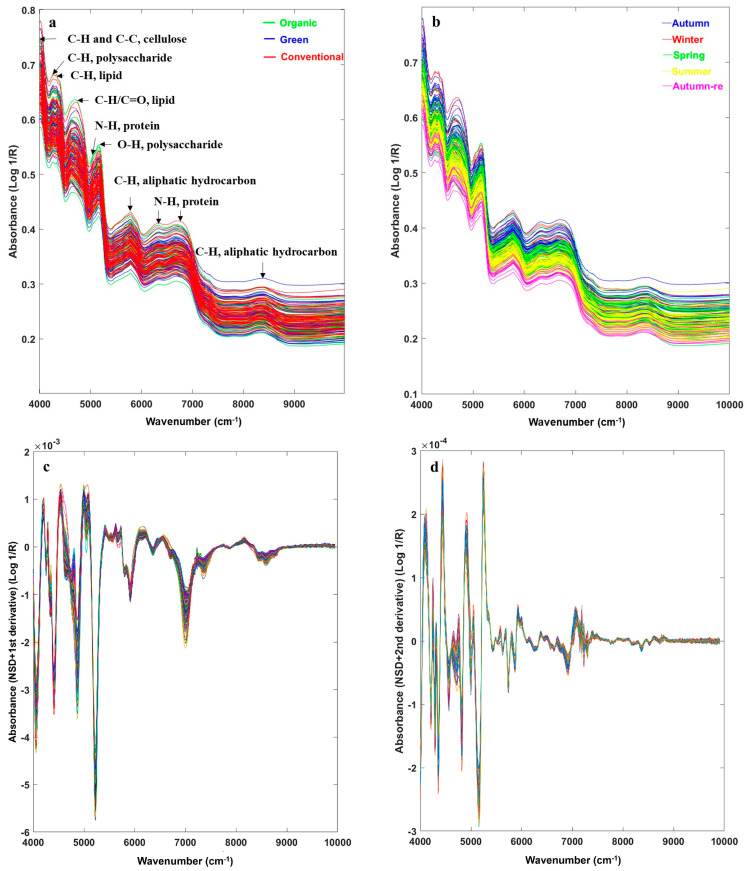
*BC* sample NIR raw spectra under different cultivation methods (**a**), different seasons (**b**), spectra treated with NSD 1st derivative (**c**), and 2nd derivative (**d**).

The rapid identification of agricultural product cultivation methods using NIRS mainly depends on distinctive nutritional composition signals in the spectra from different cultivation methods. Hydrogen-containing *BC* nutritional components primarily consist of dietary fiber, small amounts of sugars, proteins, and fats. Raw spectra of the ORG, GRE, and CON *BC*s had a similar spectral shape with significant wavenumber peaks observed at 4010 cm^−1^, 4250 cm^−1^, 4330 cm^−1^, 4670 cm^−1^, 5050 cm^−1^, 5170 cm^−1^, 5780 cm^−1^, 6350 cm^−1^, 6780 cm^−1^, and 8370 cm^−1^, corresponding to characteristic groups of the main nutrients in *BC* (Figure 4a) [23,35]. The peak at 4010 cm^−1^ corresponds to the combined frequency of C-H stretching and C-C stretching, indicating the presence of cellulose; both the peaks at 4250 cm^−1^ and 4330 cm^−1^ represent a second-order frequency doubling of C-H bending vibration in C-H groups, potentially indicating the presence of polysaccharides and lipids, respectively; the peak at 4670 cm^−1^ denotes the combination frequency of C-H stretching and C-H deformation, suggesting the presence of fats; the peak of 5050 cm^−1^ may indicate the combination frequency of N-H antisymmetric stretching and N-H in-plane bending in CONH_2_ groups of amide II, hinting at the presence of proteins; the peak at 5170 cm^−1^ represents the combination frequency of O-H stretching and HOH deformation in OH and HOH groups, indicating the existence of polysaccharides; the peak at 5780 cm^−1^ indicates the first-order frequency doubling of C-H stretching vibration in methylene groups of hydrocarbon structures; the peaks at 6350 cm^−1^ and 6780 cm^−1^ are the first-order frequency doubling of N-H stretching vibration in amide groups, indicating the presence of proteins; and the peak at 8370 cm^−1^ might be the second-order frequency doubling of C-H stretching vibration in methyl groups [23,35]. Deviations in peak positions occurred for individual *BC*s due to the influences of cultivation methods and seasons.

Overall, the *BC* raw spectra overlapped significantly (Figure 4a), as did the spectra of the three cultivation methods under each season (Figure S1). However, *BC* spectra tended to cluster under different seasons (Figure 4b), suggesting that seasons played an important role in the accumulation of nutrients in *BC*. The raw spectra of *BC*s displayed baseline drifts, band overlapping, and weak characteristic peaks (Figure 4a,b), making it challenging to directly distinguish the three cultivation methods based on their spectra. Therefore, NSD was used to reduce interferences and enhance feature signals in the NIR spectra (Figure 4c,d) [20], and the PLS-DA was utilized to build identification models for *BC* cultivation methods (Table 3).

Based on the *BC* raw spectra, the calibration model for ORG and CON *BC*s achieved an accuracy of 87.76%, and the validation accuracy was 78.79%. It was hard to visually distinguish between different *BC* cultivation methods from the first two principal component score plots of PLS-DA (Figure S2a) due to the NIR spectral data having 1557 variables and a high number of optLVs in modeling (generally more than 10, Table S2). More ORG *BC*s (*n* = 13) were wrongly classified compared to CON *BC*s (*n* = 7). In addition, summer (*n* = 7) and winter *BC*s (*n* = 5) accounted for a higher proportion of misclassified samples, suggesting possible similarities in nutritional components between ORG and CON *BC*s during these seasons. The NSD (5,5,2) pretreatment improved the model accuracies, reaching 91.84% for the calibration model and 81.82% for the validation model (Table 3 and Table S2). Misclassified samples still occurred more frequently in winter (*n* = 7) and summer (*n* = 4), possibly due to slower or faster growth in winter or summer, respectively, leading to similar accumulation rates of nutritional components and lowering cultivation methods differences. The PLS-DA model for ORG and GRE *BC*s achieved 100% calibration model accuracy and 62.96% validation model accuracy, and 60% (6/10) misclassification samples occurred in summer (*n* = 4) and winter (*n* = 2). NSD(9,9,2) preprocessing improved the validation accuracy to 70.37%. The highest number of misclassifications occurred in spring (*n* = 4), possibly indicating that NSD optimized *BC*s spectra during summer while reducing spectral information differences in spring. The PLS-DA model for GRE and CON *BC*s achieved a calibration accuracy of 96.43% and a validation accuracy of 71.43%. Overall, 81.82% (9/11) of misclassified *BC*s were from summer (*n* = 7) and winter (*n* = 2). However, the NSD preprocessing did not improve the predictive abilities of the model, with a 67.86% accuracy for the validation model, suggesting that useful spectral information might be removed when reducing disturbing signals.

Therefore, the optimal PLS-DA model for ORG and CON *BC*s showed a favorable predictive performance with an accuracy of 81.82% due to differences in their nutritional composition, while the predictive accuracies for ORG and GRE *BC*s (70.37%) and GRE and CON *BC*s (75.00%) did not achieve such good results, mainly due to the special cultivation requirements of GRE *BC*s. In general, higher *BC* misclassification rates occurred in summer and winter, possibly attributed to the dynamic physiological and biochemical responses of *BC* during these seasons. These responses were influenced by temperature and light intensity, leading to similar nutrient compositions and, thus, similar NIR spectral signals. The results confirm the importance of investigating seasonal effects on NIR spectra to build a higher-accuracy and more widely adaptable model for identifying different *BC* cultivation methods.

### 3.5. Combined IRMS and NIRS to Identify BC Cultivation Methods

Individual IRMS or NIRS PLS-DA models could not effectively identify *BC*s under different cultivation methods from the same geological origin due to the influence of fertilizer types and seasonal variations. The stable isotopic and NIR spectral differences in ORG, GRE, and CON *BC*s were comprehensively evaluated to build the identification models of IRMS combined with NIRS (IRMS-NIRS-PLS-DA) (Table 3, Tables S3 and S4).

The IRMS-NIRS-PLS-DA model for ORG and CON *BC*s showed a higher predictive accuracy of 87.88% compared to the optimal IRMS-PLS-DA (75.76%) or NIRS-PLS-DA model (81.82%). Furthermore, the NSD(5,5,2) pretreatment optimized the calibration model, further improving the accuracy to 89.80%, although the accuracy of the new validation model remained unchanged, indicating the combined PLS-DA model exhibited a more robust and predictive ability. However, the first two principal component score plots showed an overlap between ORG and CON *BC*s (Figure S2b). The number of misclassifications in the optimal NSD-PLS-DA model for ORG *BC*s (*n* = 9) was higher than for CON *BC*s (*n* = 5), possibly due to fertilizer types and seasonal effects [8,26]. The misclassified *BC*s mainly came from winter (*n* = 6) and summer (*n* = 4), possibly attributed to relative weak (winter) or strong (summer) photosynthesis, water evaporation, and transpiration, decreasing the differences in stable isotopes and nutritional compositions between ORG and CON *BC*s during these two seasons (Figure 2 and Table 3). The *δ*^15^N values still remained the most important variable for identifying between ORG and CON *BC*s according to the VIP of the NSD-PLS-DA model.

The IRMS-NIRS-PLS-DA model significantly improved the predictive performance for distinguishing between ORG and GRE *BC*s with an 81.48% accuracy, surpassing the individual IRMS or NIRS PLS-DA models (Table 3). The NSD(3,3,2) pretreatment further enhanced the accuracies of the calculation (100%) and validation (88.89%) models. Only three *BC*s (one ORG and two GRE) were misclassified, consisting of two samples from spring and one sample from winter, indicating that the combination of the two techniques effectively utilized the differential information from the four stable isotopes and NIR spectra between ORG and GRE *BC*s. The *δ*^15^N values remained the most important indicator of identifying between ORG and GRE *BC*s, as indicated by the VIP order of the optimal NSD-PLS-DA model.

For GRE and CON *BC*s, the PLS-DA model achieved calibration and validation accuracies of 90.48% and 75.00%, respectively. GRE *BC*s had a higher number of misclassifications (*n* = 13) compared to CON *BC*s (*n* = 2), mainly due to different fertilizer options available for GRE *BC*. Summer *BC*s (*n* = 8) were more prone to misclassification, further indicating relatively minor differences in stable isotopes and nutritional components between GRE and CON *BC*s in summer. After NSD(3,3,1) preprocessing of the spectra, the calibration accuracy increased to 100%, while the predictive accuracy decreased to 71.43%, indicating possible overfitting. Therefore, the optimal PLS-DA model for GRE and CON *BC*s was built using raw NIR spectra combined with IRMS. The *δ*^2^H value influenced by seasonal precipitation was the most important variable for identifying GRE and CON *BC*s based on the VIP order of the optimal PLS-DA model.

The results demonstrated that the optimal IRMS-NIRS-PLS-DA models showed better predictive abilities than individual IRMS-PLS-DA or NIRS-PLS-DA models. They could effectively identify ORG and CON *BC*s with an 87.88% predictive accuracy and the ORG and GRE *BC*s with an 88.89% predictive accuracy. However, the model for GRE and CON *BC*s with a 75.00% predictive accuracy was lower because of the fertilizer overlap between these two cultivation methods.

## 4. Conclusions

The study investigated the differences in overall and seasonal mean *δ*^13^C, *δ*^15^N, *δ*^2^H, and *δ*^18^O values among ORG, GRE, and CON *BC*s. Only overall *δ*^15^N values showed significant differences among different *BC* cultivation methods. Significant differences were observed for seasonal mean *δ*^15^N values among ORG and/or GRE and CON *BC*s during winter, spring, and summer, as well as in seasonal mean *δ*^18^O values between ORG and CON *BC*s in spring, which were primarily attributed to variations in fertilizer type, light intensity, temperature, and precipitation during different seasons. Furthermore, *BC* isotopes exhibited varying trends across seasons. Winter and spring showed relatively positive *δ*^13^C, *δ*^2^H, and *δ*^18^O values, significantly differing from those of summer, while there were no significant differences in the mean *δ*^15^N values of individual *BC* cultivation methods across seasons. These variations were most likely due to the *δ*^13^C, *δ*^2^H, and *δ*^18^O values primarily being influenced by light intensity, temperature, and precipitation, while the *δ*^15^N values were mainly affected by fertilizer type.

The IRMS-PLS-DA models could not effectively differentiate among ORG, GRE, and CON *BC*s, mainly due to similar seasonal effects and a range of different fertilizer options available for GRE *BC*s. The optimal NIRS-PLS-DA models, specifically for ORG and CON *BC*s, showed good performance, with an 81.82% predictive accuracy. The IRMS-NIRS-PLS-DA models with NSD pretreatment improved the predictive performances of ORG and CON *BC*s, with an 89.80% accuracy, and ORG and GRE *BC*s, with an 88.89% accuracy. Stable isotopes were the most useful variables for distinguishing ORG, GRE, and CON *BC*s. Although the predictive accuracy for GRE and CON *BC*s (75.00%) was lower than other groups, the results confirm that the combination of IRMS with NIRS is a robust and predictive method to identify different *BC* cultivation methods across season and interannual variations. 

The results indicate that seasonal effects vary the distribution of *δ*^13^C, *δ*^2^H, and *δ*^18^O values in short-growth *BC*s, influenced by light intensity, temperature, and precipitation. However, the impact on *δ*^15^N value is less significant, as it is primarily influenced by fertilizer type. It is challenging for individual IRMS or NIRS models to effectively identify different *BC* cultivation methods across seasons, especially for GRE *BC*. Combining IRMS and NIRS data proves to be a more feasible method to identify the three cultivation methods of *BC*s. Further studies, including seasonal and annual effects, will further validate the models’ robustness and predictive ability. 

## Figures and Tables

**Table 1:** Number of *BC* samples collected for each season and cultivation method.

Seasons	No. of Samples	ORG	GRE	CON
Autumn (September–November 2020)	22	9	4	9
Winter (December 2020–February 2021)	35	11	11	13
Spring (March–May 2021)	52	23	12	18
Summer (June–August 2021)	55	18	13	24
Autumn-re (September 2021)	11	3	4	4
Total	175	63	44	68

ORG: organic; GRE: green; CON: conventional.

**Table 2:** Stable isotope values of *BC* under different cultivation methods and seasons.

Seasons	Stable Isotopes	Cultivation Methods		
		ORG	GRE	CON
Autumn	*δ*^13^C	−28.53 ± 1.32 a	−28.17 ± 1.44 a	−28.12 ± 1.18 a
	*δ*^15^N	9.58 ± 6.05 a	7.13 ± 5.78 a	6.80 ± 8.79 a
	*δ*^2^H	−87.20 ± 9.04 a	−86.02 ± 8.91 a	−87.19 ± 9.78 a
	*δ*^18^O	19.13 ± 3.27 a	19.31 ± 2.35 a	17.98 ± 3.20 a
Winter	*δ*^13^C	−28.48 ± 1.34 a	−28.34 ± 1.54 a	−27.88 ± 1.60 a
	*δ*^15^N	10.24 ± 4.16 a	6.90 ± 4.76 ab	4.84 ± 7.46 b
	*δ*^2^H	−72.86 ± 8.01 a	−70.16 ± 8.12 a	−68.63 ± 5.73 a
	*δ*^18^O	21.63 ± 2.49 a	22.57 ± 1.62 a	21.75 ± 1.72 a
Spring	*δ*^13^C	−28.75 ± 0.81 a	−29.17 ± 0.54 a	−28.53 ± 0.86 a
	*δ*^15^N	11.82 ± 7.89 a	6.35 ± 4.43 a	1.50 ± 3.08 b
	*δ*^2^H	−74.17 ± 4.70 a	−74.87 ± 4.21 a	−72.80 ± 5.52 a
	*δ*^18^O	23.37 ± 2.61 a	21.51 ± 2.26 ab	21.65 ± 1.50 b
Summer	*δ*^13^C	−30.28 ± 1.35 a	−29.76 ± 0.65 a	−29.42 ± 1.54 a
	*δ*^15^N	9.11 ± 5.17 a	6.64 ± 5.51 ab	3.17 ± 3.33 b
	*δ*^2^H	−89.10 ± 6.47 a	−85.25 ± 8.51 a	−88.13 ± 9.71 a
	*δ*^18^O	20.28 ± 1.74 a	21.22 ± 1.33 a	20.71 ± 2.02 a
Autumn-re	*δ*^13^C	−30.14 ± 0.05 a	−29.98 ± 0.44 a	−30.45 ± 0.56 a
	*δ*^15^N	12.94 ± 5.46 a	7.29 ± 8.75 a	4.01 ± 0.31 a
	*δ*^2^H	−82.49 ± 6.02 a	−84.03 ± 6.86 a	−89.00 ± 3.55 a
	*δ*^18^O	20.14 ± 0.99 a	20.21 ± 0.79 a	19.61 ± 0.66 a

Different letters within a row indicate a significant difference for each cultivation method (*p* < 0.05). ORG: organic; GRE: green; CON: conventional.

**Table 3:** Model methods and accuracies of PLS-DA models for *BC* cultivation methods using IRMS and/or NIRS.

Instruments	Cultivation Methods	Models	Calibration Accuracy (%)	Validation Accuracy (%)
IRMS	ORG vs. CON	PLS-DA	77.55 (76/98)	75.76 (25/33)
	ORG vs. GRE	PLS-DA	71.25 (57/80)	51.85 (14/27)
	GRE vs. CON	PLS-DA	73.81 (62/84)	53.57 (15/28)
NIR	ORG vs. CON	PLS-DA	87.76 (86/98)	78.79 (26/33)
		NSD(5,5,2) ^a^-PLS-DA	91.84 (90/98)	81.82 (27/33)
	ORG vs. GRE	PLS-DA	100 (80/80)	62.96 (17/27)
		NSD(9,9,2)-PLS-DA	100 (80/80)	70.37 (19/27)
	GRE vs. CON	PLS-DA	96.43 (81/84)	71.43 (20/28)
		NSD(9,9,1)-PLS-DA	100 (84/84)	67.86 (19/28)
IRMS-NIR	ORG vs. CON	PLS-DA	83.67 (82/98)	87.88 (29/33)
		NSD(5,5,2)-PLS-DA	89.80 (88/98)	87.88 (29/33)
	ORG vs. GRE	PLS-DA	98.75 (79/80)	81.48 (22/27)
		NSD(3,3,2)-PLS-DA	100 (80/80)	88.89 (24/27)
	GRE vs. CON	PLS-DA	90.48 (76/84)	75.00 (21/28)
		NSD(3,3,1)-PLS-DA	100 (84/84)	71.43 (20/28)

^a^ The parameters (s, g, and n) in NSD were defined by: s, the number of data in one segment; g, the number of data in one gap; n, 1 or 2 is the first derivative or second derivative. ORG: organic; GRE: green; CON: conventional; NSD: Norris smoothing and derivative.

## Data Availability

The original contributions presented in the study are included in the article/Supplementary Material, further inquiries can be directed to the corresponding authors.

## Supplementary Materials

The following supporting information can be downloaded at: https://www.mdpi.com/article/10.3390/foods13081165/s1, S1: The equations of sensitivity (SE), specificity (SP), classification accuracy, and area under curve (AUC); Figure S1: *BC* raw spectra under different seasons; Figure S2: the first two principal component score plots of PLS-DA for different *BC* cultivation methods using NIR (a) and IRMS-NIR (b); Table S1: PLS-DA models of *BC* different cultivation methods using IRMS; Table S2: comparison of parameters in the NIRS-PLS-DA models with NSD preprocessing method for different BCs cultivation methods; Table S3: the *δ*^13^C, *δ*^15^N, *δ*^2^H, and *δ*^18^O values of different *BC* cultivation methods divided as the calibration and validation sets of the optimal IRMS-NIRS-PLS-DA models; Table S4: comparison of parameters in the IRMS-NIRS-PLS-DA models with NSD preprocessing method for different *BC*s cultivation methods.
