# Supplementary material for: Assessing Seasonal Effects on Identification of Cultivation Methods of Short–Growth Cycle Brassica chinensis L. Using IRMS and NIRS
Source: Foods. 2024 Apr 11;13(8):1165. doi: 10.3390/foods13081165 (PMC11049375; doi:10.3390/foods13081165)
Supplement: Supplementary file 1 [file foods-13-01165-s001.zip › foods-2936327-supplementary.pdf]

Supplementary materials

S1. The equations of sensitivity (SE), specificity (SP), classification accuracy and area under curve (AUC)

$$SE\ (\%) = \frac{TP}{TP + FN} \times 100$$

$$SP\ (\%) = \frac{TN}{TN + FP} \times 100$$

$$Accuracy\ (\%) = 1 - \frac{FP + FN}{TP + FP + TN + FN} \times 100$$

Where TP: true positive (positive samples correctly classified), TN: true negative (negative sample correctly classified), FP: false positive (positive samples incorrectly classified), FN: false negative (negative samples incorrectly classified).

Area under curve (AUC) is a receiver operating characteristic curve, which is plotted on the vertical axis of sensitivity (true positive rate) and abscissa of false positive rate (1- specificity) according to a series of different dichotomies (cut-off values or determination thresholds). The closer the AUC is to 1.0, the higher the authenticity of the method.

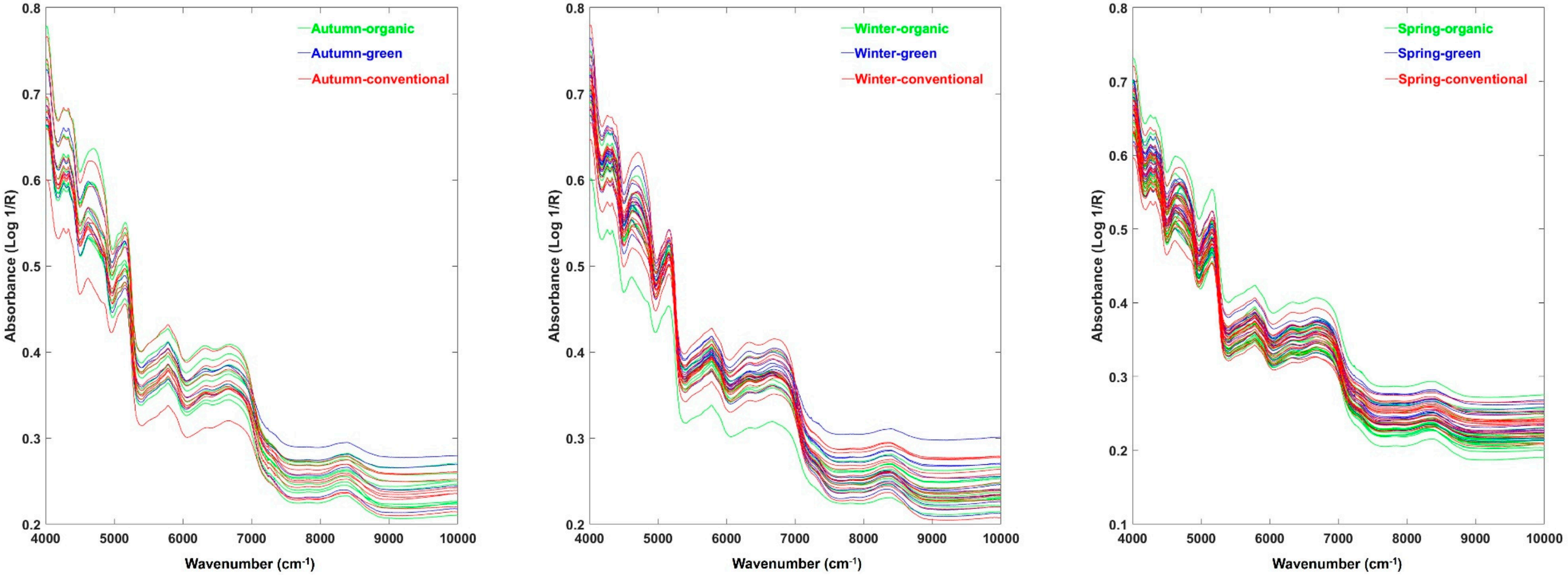
Autumn-organic
Autumn-green
Autumn-conventional
Absorbance (Log 1/R)
Wavenumber (cm-1)
Winter-organic
Winter-green
Winter-conventional
Absorbance (Log 1/R)
Wavenumber (cm-1)
Spring-organic
Spring-green
Spring-conventional
Absorbance (Log 1/R)
Wavenumber (cm-1)

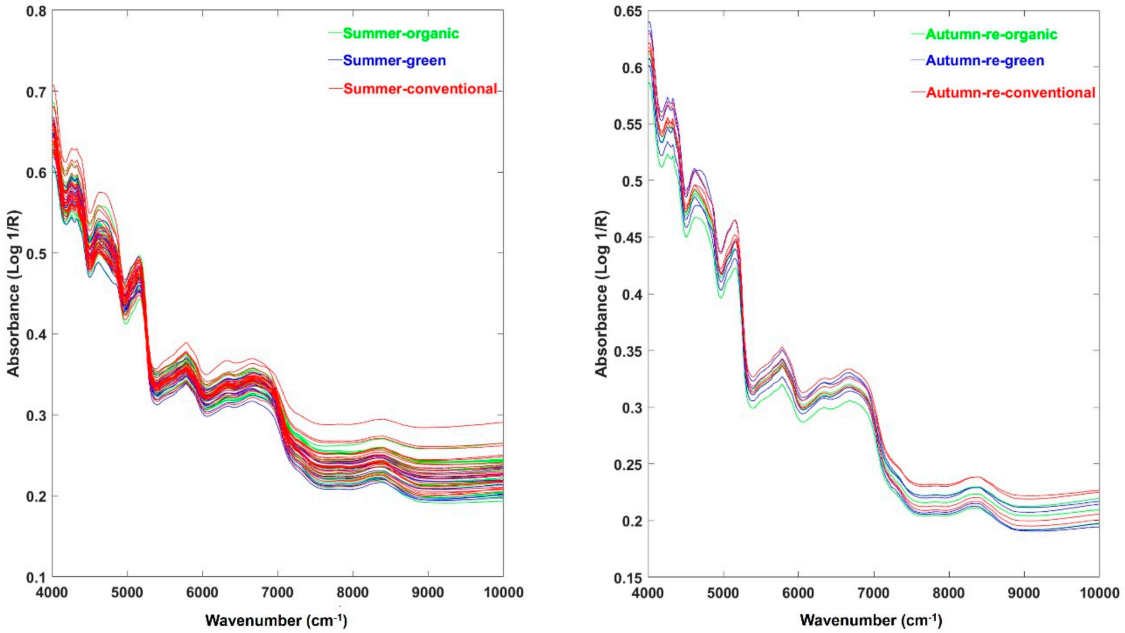

Figure S1. *BC* raw NIR spectra under different seasons

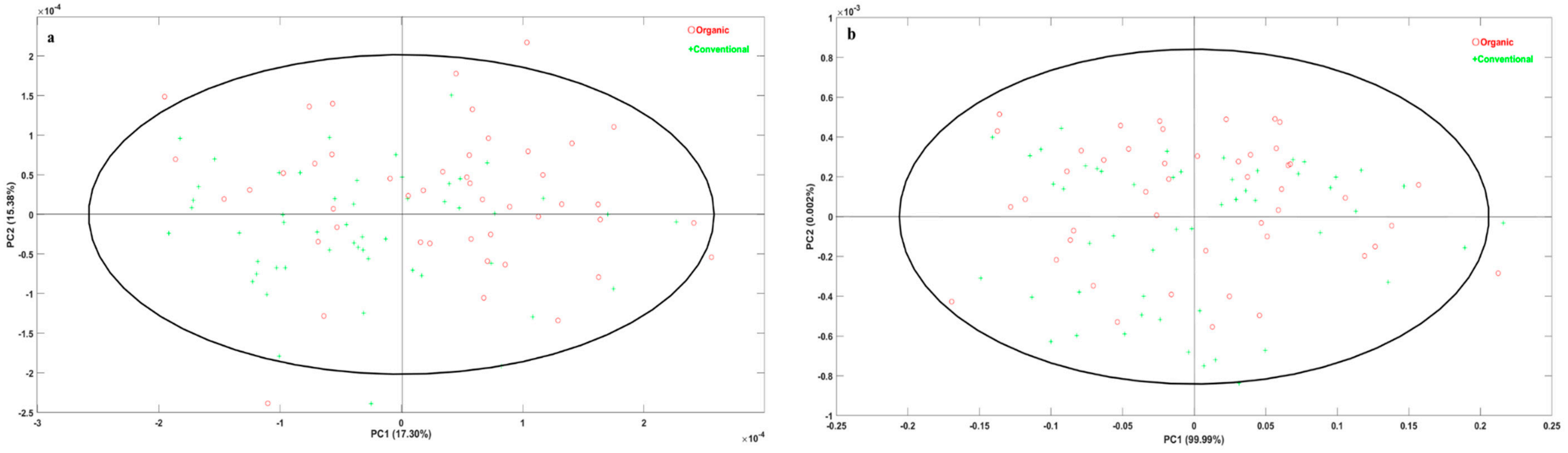

Figure S2. The first two principal component score plots of PLS-DA for different *BC* cultivation methods using NIR (a) and IRMS-NIR (b).

Table S1. PLS-DA models of *BC* different cultivation methods using IRMS

Cultivation methods	CV		Calibration set				Validation set accuracy (%)
	optLVs	Accuracy (%)	SE (%)	SP (%)	AUC (%)	accuracy (%)	
ORG *vs*. CON	2	80.45	63.64	88.89	78.68	77.55 (76/98)	75.76 (25/33)
ORG *vs*. GRE	4	61.27	88.00	43.33	70.28	71.25 (57/80)	51.85 (14/27)
GRE *vs*. CON	3	65.83	90.74	43.33	73.23	73.81 (62/84)	53.57 (15/28)

ORG: organic; GRE: green; CON: conventional; OptLVs: the optimal number of latent variables; SE: sensitivity; SP: specificity; AUC: classification error rate and area under curve.

Table S2. Comparison of parameters in the NIRS-PLS-DA models with NSD preprocessing method for different *BC*s cultivation methods.

Cultivation methods	Models	CV		Calibration set				Validation set
		optLVs	Accuracy (%)	SE (%)	SP (%)	AUC (%)	Accuracy (%)	Accuracy (%)
ORG *vs*. CON	PLS-DA	11	81.79	85.42	90.00	87.83	87.76	78.79
	NSD(3,3,1)[a]-PLS-DA	9	76.53	86.96	88.46	87.71	87.76	63.64
	NSD(3,3,2)-PLS-DA	10	73.47	100	98.08	98.94	98.98	72.73
	NSD(5,5,1)-PLS-DA	9	78.57	89.36	90.20	89.76	89.80	69.70
	**NSD(5,5,2)-PLS-DA**	**11**	**70.41**	**91.30**	**92.31**	**91.81**	**91.84**	**81.82**
	NSD(7,7,1)-PLS-DA	12	76.53	87.50	96.00	92.17	91.84	72.73
	NSD(7,7,2)-PLS-DA	22	69.39	100	100	100	100	75.76
	NSD(9,9,1)-PLS-DA	15	73.47	89.80	91.84	90.83	90.82	72.73
	NSD(9,9,2)-PLS-DA	27	69.39	100	100	100	100	66.67
ORG *vs*. GRE	PLS-DA	26	50.00	100	100	100	100	62.96
	NSD(3,3,1)-PLS-DA	16	58.75	100	100	100	100	59.26
	**NSD(3,3,2)-PLS-DA**	**14**	**51.25**	100	100	100	100	**55.56**
	NSD(5,5,1)-PLS-DA	15	51.25	96.00	96.67	95.75	96.25	66.67
	NSD(5,5,2)-PLS-DA	7	46.25	91.30	70.59	83.24	82.50	66.67
	NSD(7,7,1)-PLS-DA	24	51.25	100	100	100	100	66.67
	NSD(7,7,2)-PLS-DA	16	56.25	95.83	90.63	93.71	93.75	59.26
	NSD(9,9,1)-PLS-DA	24	58.75	100	100	100	100	66.67

	NSD(9,9,2)-PLS-DA	28	58.75	100	100	100	100	70.37
GRE *vs*. CON	**PLS-DA**	20	53.57	100	90.91	97.22	96.43	71.43
	**NSD(3,3,1)-PLS-DA**	**14**	**67.86**	**97.96**	**94.29**	**96.53**	**96.43**	**57.14**
	NSD(3,3,2)-PLS-DA	5	64.29	86.79	80.65	83.29	57.14	57.14
	NSD(5,5,1)-PLS-DA	19	59.52	100	97.14	99.00	98.81	57.14
	NSD(5,5,2)-PLS-DA	22	57.14	100	100	100	100	60.71
	NSD(7,7,1)-PLS-DA	25	58.33	100	100	100	100	60.71
	NSD(7,7,2)-PLS-DA	10	69.05	86.00	76.47	81.55	53.57	53.57
	NSD(9,9,1)-PLS-DA	23	57.14	100	100	100	100	67.86
	NSD(9,9,2)-PLS-DA	16	58.37	81.25	95.83	90.66	90.00	59.37

Table S3. The $\delta^{13}C$, $\delta^{15}N$, $\delta^{2}H$ and $\delta^{18}O$ values of different *BC* cultivation methods divided as the calibration and validation sets of the optimal IRMS-NIRS-PLS-DA models

Combination	Cultivation method	Variables	Calibration set			Validation set		
			No. of samples	Mean ± SD	Range	No. of samples	Mean ± SD	Range
ORG *vs*. CON	ORG	$\delta^{13}C$	46	-29.05±1.32	-31.17— -25.90	17	-29.51±1.44	-32.09— -26.99
		$\delta^{15}N$		+11.24±6.17	-1.28—+24.15		+8.50±6.03	-1.16—+23.13
		$\delta^{2}H$		-79.05±9.32	-100.52— -58.55		-84.28±9.89	-101.88— -66.37
		$\delta^{18}O$		+21.51±2.81	+15.41—+27.45		+21.19±3.05	+14.14—+26.53
	CON	$\delta^{13}C$	52	-28.63±1.54	-31.73— -25.10	16	-29.26±1.17	-30.69— -27.44
		$\delta^{15}N$		+4.01±5.78	-2.68—+24.44		+2.17±3.19	-3.20—+9.12
		$\delta^{2}H$		-79.94±12.11	-109.90— -61.93		-81.32±9.55	-98.57— -68.93
		$\delta^{18}O$		+20.63±2.39	+14.26—+25.70		+21.06±1.98	+17.37—+24.44
ORG *vs*. GRE	ORG	$\delta^{13}C$	50	-29.06±1.40	-32.09— -25.90	13	-29.62±1.10	-31.44— -27.99
		$\delta^{15}N$		+9.88±6.38	-1.28—+24.15		+12.87±4.98	+7.14—+20.63
		$\delta^{2}H$		-80.67±10.06	-101.88— -58.55		-79.65±8.38	-91.84— -69.03
		$\delta^{18}O$		+21.24±3.04	+14.14—+27.45		+22.12±1.91	+18.40—+24.89
	GRE	$\delta^{13}C$	30	-29.07±1.30	-31.22— -26.42	14	-29.22±0.79	-30.18— -27.63
		$\delta^{15}N$		+6.14±5.32	-1.33—+19.47		+8.01±4.69	+0.54—+15.22
		$\delta^{2}H$		-78.54±10.76	-103.83— -59.22		-78.75±6.86	-89.51— -69.88

		δ18O		+21.23±2.23	+17.01—+25.54		+21.68±1.07	+20.44—+23.33
GRE *vs*. CON	GRE	δ13C	32	-29.03±1.28	-31.22— -26.42	12	-29.36±0.72	-30.51— -28.24
		δ15N		+7.18±5.52	-1.33—+19.47		+5.55±3.97	+0.72—+13.95
		δ2H		-77.60±10.03	-103.83— -59.22		-81.29±8.12	-94.02— -70.90
		δ18O		+21.47±2.15	+17.01—+25.54		+21.12±1.22	+18.92—+23.11
	CON	δ13C	52	-28.75±1.57	-31.73— -25.10	16	-28.86±1.16	-30.69— -27.23
		δ15N		+4.21±5.81	-3.20—+24.44		+1.53±2.48	-1.84—+6.16
		δ2H		-80.43±12.29	-109.90— -61.93		-79.75±8.78	-92.56— -64.99
		δ18O		+20.64±2.44	+14.26—+25.70		+21.04±1.75	+17.37—+24.44

ORG: organic; GRE: green; CON: conventional; SD: Standard deviation.

Table S4. Comparison of parameters in the IRMS-NIRS-PLS-DA models with NSD preprocessing method for different *BC*s cultivation methods.

Combination	Models	CV		Calibration set				Validation set
		**optLVs**	**Accuracy (%)**	**SE (%)**	**SP (%)**	**AUC (%)**	**Accuracy (%)**	**Accuracy (%)**
ORG *vs*. CON	PLS-DA	12	35.71	80.43	86.54	83.71	83.67	87.88
	NSD(3,3,1)[a]-PLS-DA	12	71.43	88.37	94.55	91.96	91.84	78.79
	NSD(3,3,2)-PLS-DA	8	64.29	86.96	90.38	88.78	88.78	78.79
	NSD(5,5,1)-PLS-DA	13	73.47	86.05	94.55	91.08	90.82	78.79
	**NSD(5,5,2)-PLS-DA**	**13**	**73.47**	**89.13**	**90.38**	**89.76**	**89.80**	**87.88**
	NSD(7,7,1)-PLS-DA	13	69.39	86.05	92.73	89.86	89.80	81.82
	NSD(7,7,2)-PLS-DA	14	67.35	86.96	90.38	88.78	88.78	78.79
	NSD(9,9,1)-PLS-DA	12	71.43	90.70	83.64	86.62	86.73	84.85
	NSD(9,9,2)-PLS-DA	16	68.37	86.96	90.38	88.78	88.78	78.79
ORG *vs*. GRE	PLS-DA	25	51.25	97.87	100	98.53	98.75	81.48
	NSD(3,3,1)-PLS-DA	**10**	52.50	86.00	73.33	80.09	81.25	88.89
	**NSD(3,3,2)-PLS-DA**	16	**48.75**	**100**	**100**	**100**	**100**	**88.89**
	NSD(5,5,1)-PLS-DA	10	48.75	84.00	66.67	76.10	77.50	85.19
	NSD(5,5,2)-PLS-DA	15	52.50	94.00	100	95.45	96.25	85.19
	NSD(7,7,1)-PLS-DA	11	55.00	84.00	66.67	76.10	77.50	81.48
	NSD(7,7,2)-PLS-DA	9	60.00	90.00	70.00	82.05	82.50	81.48
	NSD(9,9,1)-PLS-DA	11	56.25	84.00	63.33	74.81	76.25	81.48

	NSD(9,9,2)-PLS-DA	9	55.00	88.00	66.67	79.20	80.00	85.19
GRE *vs*. CON	**PLS-DA**	**20**	**57.14**	**96.16**	**81.25**	**91.07**	**90.48**	**75.00**
	**NSD(3,3,1)-PLS-DA**	**26**	**63.10**	**100**	**100**	**100**	**100**	**71.43**
	NSD(3,3,2)-PLS-DA	11	51.19	98.11	96.77	97.44	97.62	57.14
	NSD(5,5,1)-PLS-DA	14	63.10	96.15	93.75	94.95	95.24	63.10
	NSD(5,5,2)-PLS-DA	12	58.33	90.57	83.87	87.22	88.10	58.33
	NSD(7,7,1)-PLS-DA	14	67.86	94.34	87.10	91.30	91.67	64.29
	NSD(7,7,2)-PLS-DA	13	60.71	88.68	70.97	81.25	82.14	71.43
	NSD(9,9,1)-PLS-DA	6	67.86	87.04	66.67	78.27	79.76	64.29
	NSD(9,9,2)-PLS-DA	11	60.71	81.13	70.97	75.72	77.38	67.86

[a] The parameters (s, g, n) in NSD were defined by: s, the number of data in one segment; g, the number of data in one gap; n, 1 or 2 is the first derivative or second derivative.

ORG: organic; GRE: green; CON: conventional; OptLVs: the optimal number of latent variables; SE: sensitivity; SP: specificity; AUC: classification error rate and area under curve.
